# Perspectives on prostate cancer: advances and pending challenges for a multidisciplinary oncological approach in South America

**DOI:** 10.1007/s11255-023-03753-4

**Published:** 2023-09-12

**Authors:** Kevin A. Diaz, Sandra Liliana Amaya, Herney Andrés García-Perdomo

**Affiliations:** 1 UROGIV Research Group, Department of Surgery, School of Medicine, Universidad del Valle, Cll 4B #36-00, Cali, Colombia; 2 Division of Urology/Urooncology, Department of Surgery, School of Medicine, Universidad del Valle, Cali, Colombia

**Keywords:** Prostate cancer, South American, Multidisciplinary treatment, Oncology outcomes, Referral centers

## Abstract

Prostate cancer is one of the tumors with the highest incidence and mortality among men worldwide, and this situation is no different in South America. However, epidemiological data are highly variable for each country and even more so than in North America. These data may be influenced by the very low rate of early detection of disease, availability of diagnostic methods, proper data collection, and limited access to specialized multidisciplinary treatment. For many South American countries, academic referral centers can only offer state-of-the-art diagnostics and multidisciplinary cancer treatment for patients who live in or can travel to large cities, so most patients are cared for by non-expert urologists with limited resources, which can have a negative impact on their prognosis and worsen oncologic outcomes. We aimed to show the clinical management of prostate cancer patients, the current advances in management, limitations present in South America, and how a multidisciplinary approach in referral cancer centers conformed of specialized urologists, medical oncologists, and mental health professionals can maximize patient outcomes.

## Introduction

Latin America is a heterogeneous region of 12 countries stretching from Colombia to the southern tip of Argentina. This region has experienced significant changes in recent decades, such as demographic distribution, increased life expectancy, development of health systems, and public health programs. These changes have increased cancer incidence throughout the region, including prostate cancer. However, despite recent improvements in healthcare, cancer mortality rates in South America (LATAM) are twice those of developed countries with higher incidences of advanced disease [1].

Prostate cancer (PCa) has undergone many changes in recent years related to understanding risk factors, better diagnostic and prognostic tools, more effective local and systemic therapy, and patient-centered care. Evidence shows that these strategies have improved oncologic outcomes and contributed to increased quality of life and survival rates for PCa. Therefore, nowadays, multimodal therapy can cure many locally advanced diseases and increase median survival in metastatic castration-resistant states [2]. Data exist for various urologic and other malignancies neoplasm that has shown that centralization of patients in high-volume hospitals and shared decision-making by a multidisciplinary team can improve oncologic outcomes, increase patient acceptance, and decrease in-hospital mortality. Therefore, it is unsurprising that several international committees and associations have recommended this process to become the standard of care [3, 4]. Nonetheless, geographical differences, lack of resources in some cases, and oncologic care by non-specialized teams have made it challenging to provide the best possible care [5, 6].

### Epidemiology

According to data from GLOBOCAN, an estimated 1.4 million new cases and 375,000 deaths worldwide from prostate cancer (PCa), being a highly prevalent disease worldwide with incidence rates ranging between 6.3 and 83, 4 per 100,000 men in different regions, with significant differences between regions due to genetic background, lifestyle, availability of screening programs, and diagnostic practices, thus it ranks as the second most common cancer and the fifth leading cause of cancer death in men for 2020; PCa is the third most frequent cancer diagnosed in LATAM after lung and breast, being the most frequent diagnosed in men, reaching one of the highest mortality rates among all cancers, being the main cause of death from cancer in some countries from South America such as Ecuador, Chile and Venezuela [7]; In Argentina, PCa is also the most frequent cancer in men, with an incidence of 42 cases per 100,000 men with mortality rate 10,3/100.000 ASR in 2020 [8]; In Colombia, PCa is the third most lethal cancer in men, after stomach and lung cancer, with an age standardized rate cancer mortality 11/100,000 men in 2017 [9]; In Peru, the reported PCa mortality rate between 2010 and 2014 was 24.1 per 100,000 men for this period, showing an increase of 15.2% compared to the previous report from 2005 to 2009, with higher rates found in the coast in contrast with the mountains and the jungle [10]; In Brazil, PCa is the most frequent tumor excluding non-melanoma skin cancers and the second cause of cancer death among those over 50 years of age; In Brazil, PCa is the most frequent tumor, excluding non-melanoma skin cancers, and the second cause of cancer death among those over 50 years of age; The National Cancer Institute (INCA) reported 15,841 deaths, equivalent to a mortality rate of 15.30 deaths per 100,000 men in 2020, and estimated 71,730 new cases of PCa for 2023–2025 equivalent a incidence rate 62.95 per 100,000 men [11, 12]; an upward trend in mortality rates from 1996 to 2006 was described),several reasons could explain this trend, such as late adoption of the PSA test, delays in cancer diagnosis and treatment, although contemporary analyses have suggested a significant reduction in mortality rates from 2006 to 2019, likely reflecting increased awareness and advances in the structure of medical care. Moreover, there are significant discrepancies between each Brazilian region and city that influence the availability of diagnostic methods and available treatments. Although contemporary analyses have suggested that decreasing trends will continue to occur in Brazil [13], in Chile, between 1955 and 1993, a slow increase in adjusted mortality rates of 1.7% per year (12.1 to 28.7/100,000 men) was reported, accelerating notably between 1993 and 1996, with increases to 12.1% annually (28.7 to 38.7/100,000 men). From 1996, a significant decrease in mortality was reported at an annual rate of 1.2% until 2019 (38.7 to 28.6/100,000 men) in all age groups. However, more significantly, at older ages [14]. Although PCa continues to be the most common cancer in men in Chile, it is not the main cause of cancer mortality since it is behind stomach, lung, and colon cancer.

While the mortality rates have declined in most high-income countries since the mid-1990s, including those in North America, Oceania, and Northern and Western Europe, probably reflecting in treatment and earlier detection through increased screening [7], during the same period, the increase in life expectancy, the consequent aging of the population and improvements in health care in LATAM inevitably results in incidence rates increased in many countries [15], initially showing a high trend in most countries. However, recent data suggest a decreasing trend in Brazil, Colombia, Chile, and Uruguay, suggesting an improvement in managing the disease [17]. Nevertheless, the cancer mortality rates in LATAM are almost double those of high-income countries In North America (14.2 vs. 8.4) [1, 16].

Although most of the population in South America has basic health coverage, significant disparities persist between health systems in different countries, even between different regions within each country [17]. There is a significant discrepancy in resources, early diagnosis techniques, and access to specialized care between public and private health systems, which, together with geographic disparities and low socioeconomic status, can create disadvantages, increase mortality rates, and worsen oncological outcomes in these patients [18]. Therefore, health systems in LATAM must design prevention policies, early diagnosis, increased access to specialized care, and effective treatment to reduce these disadvantages.

### Screening and diagnosis

PSA for early detection of PCa was widely adopted in the late 1980s in the US. However, the United States Preventive Services Task Force (USPSTF) issued a recommendation against cancer screening in 2012 due to the limitations and risks of this assay and the unproven survival benefit during this period. Therefore, it took many years for a high-quality study to demonstrate a survival benefit; however, the number of men needed to be invited for screening to prevent one PCa death was 570 at 16 years [19]. In 2018, the task force revised its previous statement. It changed the PSA screening recommendation to an individualized, shared decision between patients and physicians for men at increased risk of prostate cancer aged 50 to 69 years and with a life expectancy of at least 10 to 15 years [20], seeking a balance between the beneficial effect of early diagnosis and the hazards of overdiagnosis and overtreatment.

PSA screening is not extensively used in LATAM, and there are few programs governmental to early diagnosis of PCa, being a majority of these initiatives are isolated campaigns and programs promoted by specific groups or academic centers. Only a few studies for the early detection of prostate cancer have been conducted in LATAM. In Argentina, 2686 men 24–96 years old (mean age 63.4 years) were screened with Digital Rectal Examination DRE and PSA and/or Transrectal Ultrasound (TRUS) at the Hospital de Clinicas Jose de San Martin. Of the 576 with abnormal results, 285 underwent TRUS, and 65 prostate cancer cases were identified (detection rate 2.4%) [16]. In Brazil, the Barretos Cancer Hospital screened 17,571 men 45 years and older for prostate cancer (PSA and DRE) from January 2004 to December 2007, diagnosed 652 prostate cancer cases, mostly with localized disease (T1–T2 93.4% and Gleason score of ≥ 7 was 32.5%), with PPV 70.9% for the 7.1% of men with both suspicious DRE findings and a PSA level of ≥ 4.0 ng/mL [21]. In Monterrey (Mexico), a screening program screened 973 men 40 years and older using PSA and DRE from 2004 to 2006, prostate biopsy was recommended to 125, but only 55 (44%) of them underwent a transrectal biopsy was diagnosed with prostate cancer in 15 men, mainly with Gleason scores ≥ 7 [22]. Another more contemporary study involving 1672 men, according to the latest available National Comprehensive Cancer Network (NCCN) prostate cancer early detection guidelines and evaluated the clinical utility of the Prostate Cancer Prevention Risk Collaborative Biopsy Group (PBCG) and Prostate Cancer Prevention Risk Calculator Test (PCPTRC) 2.0 to guide the prostate biopsy decision, 687 patients underwent ultrasound or MRI/US-guided prostate biopsy, 135 (31.17%) patients were diagnosed with high-grade prostate cancer, 63 (14.54%) with low grade, and 235 (54.27%) with negative prostate biopsy results, although the use of both models to guide the prostate biopsy decision did not show a statistical difference between the detection rates of high-grade PCa, but did achieve a significant difference in the reduction of total and unnecessary biopsies. Therefore, it demonstrated that both risk calculators could be used to improve prostate cancer screening programs [23].

European Association of Urology (EAU) guidelines currently recommend the use of prostate Magnetic Resonance Imaging (MRI) and a risk calculator to guide biopsy decisions and limit unnecessary procedures [24, 25]. Even though available biomarker tests, such as the Prostate Health Index, 4K score, SelectMdx, ExoDx Prostate, and sentinel tests might improve prostate cancer detection, reduce the diagnosis of clinically insignificant cancers, and reduce unnecessary biopsies when used in combination with MRI [2]. However, more studies are needed to validate their efficacy [24]. Currently, there are minimal data to recommend the application of these markers in routine screening programs [24, 26]. The availability of these methods in developing countries in Latin America, such as Mexico, Argentina, Colombia, and Brazil, is limited mainly by their costs, which cannot be borne by most major health systems, particularly in the public sector, making them not cost-effective for many health systems however, risk calculator such as PCPTRC 2.0 and PBCG are easy to implement tools that can improve the benefit of early diagnosis and decrease the danger of overdiagnosis [23].

### Genetic testing for prostate cancer

Increasing evidence supports a predisposition to PCa caused by alterations in DNA repair genes. Breast cancer susceptibility proteins (BRCA) 1 and 2 may drive the development of the disease and more aggressive forms. These mutations occur in approximately 0.2% to 0.3% of the general population. However, they are found in 5.3% and 0.9% of men with metastatic prostate cancer without association with a family history, with a reported incidence of 11.8%, significantly exceeding the prevalence in the general population and men with localized prostate cancer with increased risk of prostate cancer (RR 2.5, 1.6 to 3.8 CI 95%) [2, 27, 28]; being independent predictors of metastasis and worse PCa-specific survival [24, 29]. Screening is especially relevant in men with a known pathogenic BRCA variant, and early diagnostic tools should be considered. There are few studies analyzing germline mutations in Latin American men; one conducted outside of LATAM (San Antonio, Texas) evaluated 1515 Hispanic men diagnosed with PCa or with a first-degree family history using the Color Genomics platform and reported that Hispanic men with a personal or family history of PCa carry mutations in inherited cancer genes at a significant rate [30]. Therefore, germline tests for BRCA1/2, ATM, and MMR are recommended for high-risk PCa and the metastatic stage, so these could be considered in South America. However, they are poorly available and limited mainly by their costs.

### Prostate-specific membrane antigen (PSMA) PET SCAN

PSMA is a type II transmembrane glycoprotein that is highly expressed in almost all prostate cancer cells (90–95%), and it is used to improve the accuracy of conventional imaging [31]. Hofman et al. compared conventional imaging staging with CT and bone scan versus PET-CT with gallium-68 PSMA-11. He reported that the accuracy of PET/CT with 68 Ga-PSMA was 27% (95% CI 23 to 31) higher than that of CT and bone scan (92% [88–95] vs. 65% [60–69]). In addition, PET/CT scanning resulted in more frequent treatment changes compared to conventional imaging, fewer equivocal findings (7% [4–13] vs. 23% [17–31]), and less radiation exposure [32]. In addition, Chen et al. found that PSMA PET and MRI used in combination (PET/MR) prior to RP performed better than MRI in detecting clinically significant CaP, with improved sensitivity (89% vs. 76%) without sacrificing specificity (96% vs. 88%). This improvement in accuracy was particularly evident for lesions classified as PI-RADS 3 [33]. Roach et al. evaluated changes in planned management before and after PET/CT with PSMA in intermediate- and high-risk patients referred for primary staging compared to conventional staging. This study revealed unsuspected disease in the prostate bed in 27% of patients, locoregional lymph nodes in 39%, and distant metastatic disease in 16%, and management changes occurred in 21% [34]. A current review reported that PET/MR has superior accuracy for the detection of biochemical cancer recurrence (BCR) and oligometastatic disease, even in patients with very low PSA levels than conventional methods and other PET/CT studies, with detection rates of 44% (0.2 ng/mL) and 72.7% (0.2–0.5 ng/mL) [35]. These PSMA-based radiotracers have shown data suggesting a greater diagnostic value in combination with CT/MR imaging of PCa, with increased accuracy for primary staging, biochemical/relapse restaging, radiotherapy planning, and systemic therapy planning. However, the still low availability of this method limits the substantial advantages offered by this diagnostic modality over conventional diagnosis.

### Advances in the treatment of localized disease

Advances in the diagnosis and treatment of PCa have improved the ability of physicians to classify patients according to their risk and propose treatment based on cancer prognosis and patient preferences; radical prostatectomy (RP) and radiotherapy are considered the standard treatments for stage I–III PCa [36]. The last two decades have seen a transition from open RP to laparoscopic radical prostatectomy (LRP) and, currently, a growing trend in the adoption of “robotic-assisted” radical prostatectomy (RARP) with excellent results in skilled hands that suggest more efficiency at preserving the erectile function and urinary continence with perioperative morbidity advantage over RP but higher cost [37, 38]. However, a Cochrane review comparing RARP or LRP versus open RP found no significant differences between oncologic, urinary, and sexual function outcomes for each technique. RARP and LRP significantly improved the length of hospital stay and blood transfusion rates over open RP [38]. Then, no surgical approach can be recommended over another, and oncologic and functional outcomes remain closely tied to surgeon experience and high-volume hospital care [24, 39].

Since 2009, there have been many public hospitals in LATAM that have acquired the Da Vinci robotic system; even fewer institutions have transitional or definitive urology robotic-assisted surgery programs, which is hardly available to the public health system, and there is little data on robotic-assisted surgery [40]. Brazil is one of the few countries with robotic platforms, with 75 available, but only one is accessible to the public system. A retrospective study collected data from 58 patients with prostate adenocarcinoma undergoing RARP at the Monte Klinikum Hospital in Fortaleza-CE, Brazil, reported that robotic-assisted radical prostatectomy performed by trained surgeons is feasible and safe with satisfactory functional and oncologic outcomes without increasing complication rates. However, the number of patients treated was deficient [41]. Therefore, the adoption and development of robotic surgery in other South American countries have been limited by high costs, lack of equipment infrastructure, and lack of experience in this field.

### Triplet therapy in metastatic castration-sensitive prostate cancer (mHSPC)

The landscape of treatment options and recommendations for patients with mHSPC has changed significantly in recent years, with the emergence of evidence demonstrating how the intensification of the survival benefit of androgen signaling blockade and combination therapies improved clinical outcomes for men with mHSPC. The ARASENS trial involved 1306 patients; 86.1% had metastatic disease at diagnosis (bone or visceral metastases), and in which a significantly more significant improvement in overall survival was reported among patients who received combination therapy with darolutamide, ADT, and docetaxel than among those who received androgen-deprivation therapy and docetaxel alone. In addition, there was a 32% reduction in the risk of death (HR 0.68; 95% Cl 0.57 to 0.80) with a similar frequency of adverse effects [42]. In addition, the PEACE1 enrolled 1173 patients with histologically or cytologically confirmed de novo metastatic prostate adenocarcinoma and evaluated ADT and docetaxel ± radiotherapy and ADT, docetaxel, abiraterone ± radiotherapy, reported overall survival (0.82, 95% CI 0.69 to 0.98) and increased radiographic progression-free survival (rPFS) (HR 0. 54, 99.9% CI 0.41 to 0.71) for the group receiving abiraterone compared to patients who did not. It demonstrated that the combination of ADT, docetaxel, and abiraterone in castration-sensitive metastatic prostate cancer improved overall survival and rPFS without increasing rates of severe or medically significant adverse events (neutropenia, febrile neutropenia, fatigue, or neuropathy) [43]. Subsequently, three meta-analyses confirmed that intensification of initial treatment in mHSPC patients with androgen pathway inhibition (API) in addition to docetaxel and ADT could improve clinical outcomes [44–46].

Emerging results support a clear survival advantage, an improvement in rPFS in favor of triple therapy over other available regimens, with no increase in severe or medically significant adverse events. Therefore, health authorities should contribute to increasing the availability and accessibility of these combination therapies, as they have been shown to impact overall survival substantially and are superior to conventional regimens.

### Advanced disease: androgen-deprivation therapy, nonmetastatic castration-resistant PCa (nmCRPCa), and metastatic disease

Androgen-deprivation therapy (ADT) blocks the production of testosterone and other male hormones, preventing them from feeding prostate cancer cells. It is recommended in the treatment of advanced and/or metastatic PCa. However, these treatment options are not curative, and the disease progresses to the castration-resistant phenotype over time [36]. Until 1990, only bilateral orchiectomy and LHRH analogs were available for hormonal treatment. However, in the last 25 years, the overwhelming clinical success of androgen receptor (AR)-targeted agents have demonstrated significant benefits in metastasis-free survival (MFS) and improved survival in nmCRPCa (M0). The use of enzalutamide (PROSPER trial) more than doubled the MFS vs. placebo (median: 36.6 vs. 14.7 months, HR = 0.29) [47]. Darolutamide also demonstrated a significant increase in MFS compared to placebo (median: 40.4 vs. 18.4 months; HR = 0.41), and Apalutamide showed an improvement in MFS compared to placebo (median: 40.5 vs. 16.2 months; hazard ratio [HR] = 0.28) [48, 49]. However, the combination of androgen biosynthesis inhibition (Abiraterone) with AR inhibition (Enzalutamide) has not been shown to improve progression-free survival [50]. Therefore, it is recommended for inclusion as part of first-line treatment for nmCRPCa when the PSA DT is equal to or less than ten months, displacing non-steroidal antiandrogens such as nilutamide, flutamide, and bicalutamide or corticosteroids as second-line therapy.

Currently, poly-ADP ribose polymerase (PARP) inhibitor therapy for men with mCRPC whose prostate cancer harbors DNA repair mutations has prolonged survival. Olaparib (PROfound trial) demonstrated significant improvement among men with BRCA1/2 or ATM mutations [51]. Rucaparib has been approved for patients with deleterious BRCA mutations and reported a PSA response rate in 54% of treated patients [52]. Therefore, the success of PARP inhibitor therapy has led to a strong recommendation for genetic testing of patients with metastatic mCRPC or patients diagnosed with mCRPC with demonstrated mutations in DNA repair genes in their family members [24]. However, implementing these international recommendations in South America is limited by financial barriers, lack of access to new drugs approved for PCa, and lack of specialized healthcare providers in advanced disease.

### Multidisciplinary approach for prostate care

As early as the 1990s, it became clear that multidisciplinary care is an effective way to provide optimal cancer care based on interdisciplinary discussions of available scientific and clinical data by individual specialists and experts. The uro-oncology multidisciplinary discussion team (MTD) has an increasingly prominent role in cancer care, being the recommended care practice in most international guidelines, including prostate cancer. This approach has been shown in several studies to positively impact many urogenital diseases in multiple dimensions for the patient. An Italian study analyzed data from 2260 multidisciplinary clinics between March 2005 and March 2011 and found that 11% of drug therapies (ADT) prescribed outside the center had to be discontinued in the multidisciplinary clinic, and 6% of indications formulated in the cancer center changed during the MDT [53]. Another study analyzed 135/195 eligible patients over 2 years, reporting high patient satisfaction levels and confidence in the final treatment decision [54]. There is evidence that in PCa in the multidisciplinary approach can improve care processes, shorten the time to receipt of initial therapy, increase the likelihood of receipt of multimodality therapy, increase the likelihood of adherence to international guidelines, increase patient satisfaction, and reduce costs. There is evidence that the multidisciplinary approach in PCa can improve care processes, shorten the time to receipt of initial therapy, increase the likelihood of receiving multimodality therapy, increase the likelihood of adherence to international guidelines, increase patient satisfaction, and reduce costs [55]. Therefore, this multidisciplinary approach may contribute to increased quality of life and survival rates for patients with PCa.

## Conclusion

The last 2 decades have seen tremendous advances in prostate cancer treatment, with a significant impact of screening on prostate cancer diagnosis and mortality and molecular tools for risk stratification and the development of new therapies to prolong overall survival, so oncology programs focused on prevention, early diagnosis and access to effective treatment should be developed to reduce the burden of PCa for South America.

Although there are many barriers as high costs, lack of equipment infrastructure, and lack of specialized team care, that do not allow the extrapolation of all international recommendations for the management of PC, the development of structured multidisciplinary care that contributes to maximizing outcomes and increase patient quality of life should be encouraged.
